# TAK1 Inhibitor Enhances the Therapeutic Treatment for Glioblastoma

**DOI:** 10.3390/cancers13010041

**Published:** 2020-12-25

**Authors:** Michela Campolo, Marika Lanza, Giovanna Casili, Irene Paterniti, Alessia Filippone, Maria Caffo, Salvatore M. Cardali, Ivana Puliafito, Cristina Colarossi, Gabriele Raciti, Salvatore Cuzzocrea, Emanuela Esposito

**Affiliations:** 1 Department of Chemical, Biological, Pharmaceutical and Environmental Sciences, University of Messina, 98122 Messina, Italy; michela.campolo@unime.it (M.C.); mlanza@unime.it (M.L.); gcasili@unime.it (G.C.); ipaterniti@unime.it (I.P.); afilippone@unime.it (A.F.); salvator@unime.it (S.C.); 2 Department of Biomedical and Dental Sciences and Morpho-Functional Imaging, Unit of Neurosurgery, University of Messina, 98122 Messina, Italy; mariella.caffo@unime.it (M.C.); salvatore.cardali@unime.it (S.M.C.); 3 Istituto Oncologico del Mediterraneo, Via Penninazzo 7, 95029 Viagrande, Italy; ivana.puliafito@grupposamed.com (I.P.); cristina.colarossi@grupposamed.com (C.C.); 4 IOM Ricerca S.r.l., Via Penninazzo 11, 95029 Viagrande, Italy; gabriele.raciti@grupposamed.com; 5 Department of Pharmacological and Physiological Sciences, Saint Louis University School of Medicine, Saint Louis, MO 63104, USA

**Keywords:** TAK1, temozolomide, glioblastoma, cell lines, patients

## Abstract

**Simple Summary:**

Most patients with glioblastoma (GBM) develop recurrent diseases which can be treated with different approaches. Given the aggressive and resilient nature of GBM, continued efforts to better understand GBM pathophysiology are required to discover novel targets for future therapy. The aim of this study was to investigate novel therapy to associate to temozolomide (TMZ) regimens. This study indicated the important role of 5Z-7-oxozeaenol in increasing the sensitivity of glioblastoma cells to chemotherapy, proposing itself as an effective coadjuvant to current chemotherapeutic regimens. Moreover, it denoted the incessant involvement of mitogen-activated protein kinase (MAPKs) in tumorigenesis following chemotherapy.

**Abstract:**

Glioblastoma (GBM) is a brain tumor characterized by poor therapeutic response and overall survival. Despite relevant progress in conventional treatments represented by the clinical use of temozolomide (TMZ), a combination of approaches might be a possible future direction for treating GBM. Transforming growth factor-beta-activated kinase-1 (TAK1) is an essential component in genotoxic stresses-induced NF-κB-activation and mitogen-activated protein kinase (MAPK)-pathways; however, the role of TAK1 in GBM-chemoresistance remains unknown. This study aimed to verify, in GBM human cell lines, in an in vivo U87-xenograft model and in TMZ-treated-patients, the effect of TAK1 inhibition on the sensitivity of GBM cells to chemotherapy. In vitro model, using GBM cell lines, showed that 5Z-7-oxozeaenol augmented the cytotoxic effects of TMZ, blocking TMZ-induced NF-κB-activation, reducing DNA-damage and enhancing TMZ-induced apoptosis in GMB cell lines. We showed a reduction in tumor burden as well as tumor volume in the xenograft model following the treatment with 5Z-7-oxozaenol associated with TMZ. Our results showed a significant up-regulation in TAK1, p-p38, p-JNK and NF-κB in glioblastoma TMZ-treated-patients and denoted the role of 5Z-7-oxozeaenol in increasing the sensitivity of GBM cells to chemotherapy, proving to be an effective coadjuvant to current GBM chemotherapeutic regimens, suggesting a new option for therapeutic treatment of GBM.

## 1. Introduction

Glioblastomas represent the malignant brain tumor commonly stated with the worst prognosis. GBM constitutes 45% of all malignant central nervous system (CNS) tumors and 80% of all primary malignant CNS tumors. The average annual age-adjusted incidence rate is of 3.19 cases per 100,000 person-years commonly higher in men than in women and two times more common in white than in black people. Given its aggressiveness and rapid outspread in all its forms, the tumor scenario is expected to be worse than the last decade [1,2]. GBM is characterized by diffuse infiltration of the brain tissue also surrounding the bulk of the tumor, associated with chromosomal and genetic mutations that determine the uncontrolled growth and tumor cell chemo-resistance [3,4]. Despite new therapeutic advances aimed to investigate multiple oncogenic signaling aberrations associated with this tumor, the machineries of GBM onset and progression are still widely obscure. GBM is usually treated through neoplastic mass removal, in order to obtain the most optimal cerebral decompression, followed by radiotherapy and chemotherapy, which should improve and prolong overall survival. [1]. Indeed, the 5-year survival rate for patients with GBM remains low and novel therapies are needed [5].

The therapeutic standard for patients with GBM is temozolomide (TMZ), an orally alkylating agent considered as a first-line drug for GBM treatment, that is reported to increase the median survival of affected patients [6,7,8].

Unfortunately, around 50% of TMZ treated patients do not respond to this therapy, due to mainly to the over-expression of O^6^-methylguanine methyl-transferase and lack of a DNA repair machinery in GMB cancer cells [9]. Advances in research concerning this type of brain tumor are limited because of several resistance mechanisms in response to a single inhibitor [1,6]; this could suggest that combination approaches, involving standard chemotherapy and pathway inhibitors, might be a possible future direction for treating GBM. Molecular analysis of GBM revealed enrichment of knew nuclear factor κB (NF-κB), target genes showing that NF-κB inhibition reduced tumor proliferation and extended cell survival [10]. The GBM is divided into four subtypes: pro-neural, mesenchymal, classical, and neural. The mesenchymal subtype, both with primary and recurrent tumors, tends to have the worst survival rates compared to other subtypes. Specifically, the genetic abnormalities mainly involve neurofibromatosis 1(NF1) mutations and NF-κB transcriptional programs that represent the main drivers of acquiring the mesenchymal-signature and these are expressed as a consequence of higher overall necrosis and associated inflammatory infiltrates [11,12].

The role of tumor growth factor (TGF)-β–mediated signaling has paradoxical protumor effects in different cancer types, including GBM [13]. Moreover, although TGF-β typically represses NF-κB signaling in normal cells, recent evidence suggests an aberrant ability of TGF-β signaling to activate NF-κB signaling in several different cancers [14,15] as a means of promoting malignant tumor cell phenotypes. In fact, recently it has been shown that a source of NF-κB activation in GBM involves the TGF-β/transforming growth factor beta-activated kinase 1 (TAK1) signaling axis [16].

TAK1 is a member of the mitogen-activated protein kinase (MAPK) family and was found to function as an intermediate for kappaB Kinase (IKK) activation in reaction to multiple stimuli, playing a key role in the immune response [17,18,19,20,21]; the activated TAK1 in turn stimulates NF-κB and MAPK pathways in response to genotoxic stresses [22,23,24,25,26,27]. Moreover, it has been shown that TAK1 inhibition, mediated by RNAi-silencing or a TAK1 inhibitor, significantly reduced NF-κB expression, sensitizing pancreatic cancer cells to gemcitabine-induced cell-death [28]. Additionally, in breast cancer, TAK1 has been recognized as a potential target to trigger increased efficiency of topoisomerase inhibitors [29]. Moreover, TAK1 plays a key role in tumor-associated macrophages, promoting non-small cell lung carcinoma (NSCLC) growth and apoptosis in KRAS-dependent colon cancer [30,31]. In addition, among brain tumors, TAK1 inhibition considerably increased the sensitivity of neuroblastoma cells to chemotherapy [32]. The balance between pro-apoptotic and pro-survival pathways is essential to determine the effect of chemotherapy. Therefore, given that NF-κB is one of the principal survival signals activated in cancer cells, we could affirm that inhibition of TAK1 activation could alter this balance, sensitizing cells to chemotherapy. Since the MAPK and NF-κB pathways are highly mutated or deregulated in GBM, we hypothesized that the association of TMZ with an inhibitor of TAK1 (5Z-7-oxozeaenol) could enhance the sensitivity of glioblastoma cells to chemotherapy treatment with TMZ; a second step, aimed to validate the involvement of TAK1 in GMB patients treated with TMZ.

## 2. Results

### 2.1. Preliminary Study to Choose the Effective Dose with High Toxicity 

**Figure 1:**
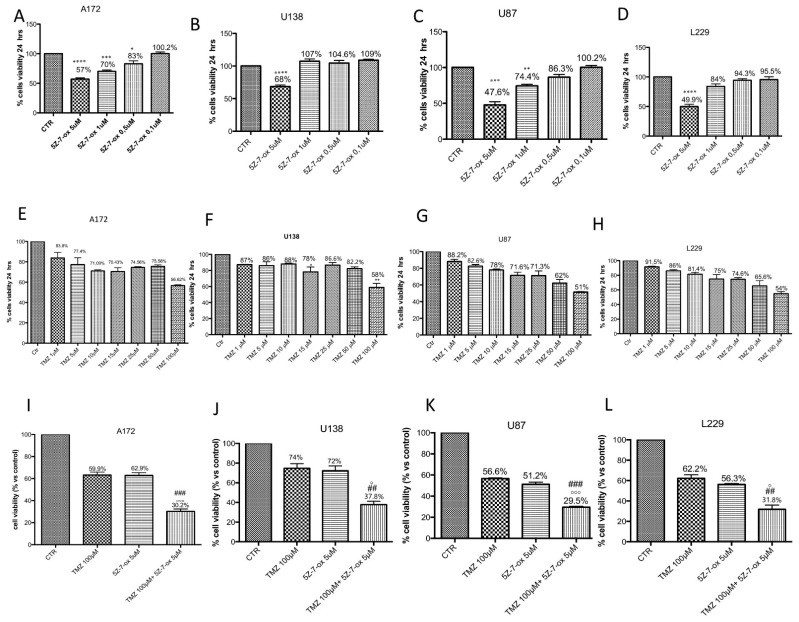
Effect of 5Z-7-ox and temozolomide (TMZ) on survival of glioblastoma cell lines. Cell viability was evaluated using MTT tetrazolium dye and quantified by measurement of optical density at 550 nm (OD550) in glioblastoma cell lines: A172, U138, U87 and L229. Cell death was significantly higher in groups treated with 5Z-7-ox at 5 μM (**A**–**D**) and TMZ at 100 μM (**E**–**H**) with a mortality percentage at least 50%. (**A**) **** *p* < 0.0001 vs. control (CTR); *** *p* < 0.001 vs. CTR; * *p* < 0.05 vs. CTR. (**B**) **** *p* < 0.0001 vs. CTR. (**C**) *** *p* < 0.001 vs. CTR, ** *p* < 0.01 vs. CTR. (**D**) **** *p* < 0.0001 vs. CTR. (**F**) ** *p* < 0.01 vs. CTR. Moreover, cell death was significantly higher in groups treated with 5Z-7-ox at 5 μM and TMZ at 100 μM (**I**–**L,** respectively). However, the association between 5Z-7-Ox 5 μM and TMZ 100 μM enhanced the cytotoxic effect of TMZ (50%) (**C**) on cell lines. Data are representative of at least three independent experiments. Data are representative of at least three independent experiments. (**I**) (A172) ### *p* < 0.001 vs. TMZ, °°° *p* < 0.001 vs. 5Z-7-ox; (**J**) (U138) ## *p* < 0.01 vs. TMZ, ° *p* < 0.05 vs. 5Z-7-ox; (**K**) (U87) ### *p* < 0.001 vs. TMZ, °°° *p* < 0.001 vs. 5Z-7-ox; (**L**) (L229) ## *p* < 0.01 vs. TMZ, ° *p* < 0.05 vs. 5Z-7-ox.

A172 (Figure 1A–E), U138 (Figure 1B–F), U87 (Figure 1C–G) and L229 (Figure 1D–H) cell viability was assessed following 24 h of treatments with different concentrations of TMZ (1 μM to 100 μM) and 5Z-7-oxozeaenol (0.1 μM to 5 μM) using the 3-(4,5-dimethylthiazol-2-yl)-2,5-diphenyltetrazolium bromide (MTT) assay and light microscopic observation. The best concentrations able to markedly reduce cell viability (±50%) were 5 μM for 5Z-7-oxozeaenol (Figure 1A, F(4;10;14) = 43.23, *p* < 0.001; Figure 1B, F(4;10;14) = 43.23, *p* < 0.001; Figure 1C, F(4;10;14) =52.82, *p* < 0.001; Figure 1D, F(4;10;14) = 33.06, *p* < 0.001) and 100 μM for TMZ (Figure 1E, F(7;16;23) = 994.3, *p* < 0.001; Figure 1F, F(7;16;23) = 10.68, *p* < 0.001; Figure 1G, F(7;16;23) =24.09, *p* < 0.001; Figure 1H, F(7;16;23) = 15.17, *p* < 0.001) in all cell lines.

### 2.2. TAK1 Inhibition Significantly Enhances the Cytotoxic Effect of TMZ on Glioblastoma Cell Lines

A172 (Figure 1I), U138 (Figure 1J), U87 (Figure 1K) and L-229 (Figure 1L) cell viability was assessed following 24 h of treatments with TMZ (100 μM) in association with 5Z-7-Ox (5 μM) using the MTT assay and light microscopic observation. The association of 5Z-7-Ox with TMZ enhanced the cytotoxic effect of single treatments on glioblastoma cell lines (Figure 1I, F(3:8;11) = 178.56, *p* < 0.001; Figure 1J, F(3;8;11) = 44.59, *p* < 0.001; Figure 1K, F(3;8;11) = 674.1, *p* < 0.001, Figure 1L, F(3;8;11) = 105.4, *p* < 0.001, compared to the single treatments.

### 2.3. TAK1 Inhibition Enhances TMZ- Induced Apoptosis

**Figure 2:**
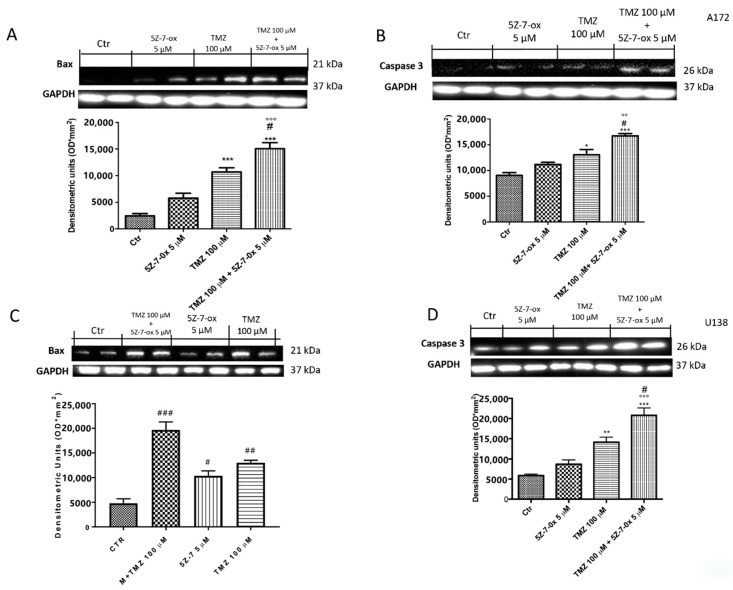
Effect of 5Z-7-ox and TMZ on Bax and Caspase 3 expression in glioblastoma cells. The association between 5Z-7-ox and TMZ decrease significantly increased the expression of Bax and caspase 3 in A172 and U138 cell lines (**A**–**D,** respectively). (**A**) *** *p* < 0.001 vs. CTR; # p < 0.001 vs. TMZ and °°° *p* < 0.001 vs. 5Z-7-ox; (**B**) * *p* < 0.001 vs. CTR, *** *p* < 0.05 vs. CTR., # *p* < 0.001 vs. TMZ and °° *p* < 0.01 vs. 5Z-7-ox. The association of 5Z-7-ox with TMZ significantly modulated their expression much more of single components (**A**–**D,** respectively). Data are representative of at least three independent experiments. (**C**) # *p* < 0.001 vs. CTR, ### *p* < 0.001 vs. CTR, ## *p* < 0.01 vs. CTR; (**D**) ** *p* < 0.01 vs. CTR, *** *p* < 0.001 vs. CTR, # *p* < 0.05 vs. TMZ and °°° *p* < 0.001 vs. 5Z-7-ox. Original blots are shown in Figures S1 and S2.

To verify if TAK1 inhibitor improved efficacy of chemotherapeutic agents to induce apoptosis in glioblastoma cell lines, we assessed Western blot analyses of pro-apoptotic proteins such as Bax and caspase 3. Combinatory treatment of TMZ and 5Z-7-oxozeaenol considerably increased the expression of both Bax and caspase 3 in glioblastoma cell lines (Figure 2A, F(3:4;70) = 158.6, *p* < 0.001; Figure 2B, F(3;8;11) = 24.81, *p* < 0.001; Figure 2C, F(3;4;7) =108.6, *p* < 0.001; Figure 2D, F(3;4;7) = 129.4, *p* < 0.001; see densitometry analyses), compared to single components.

### 2.4. TAK1 Inhibition Enhances TMZ-Induced DNA Damage

**Figure 3:**
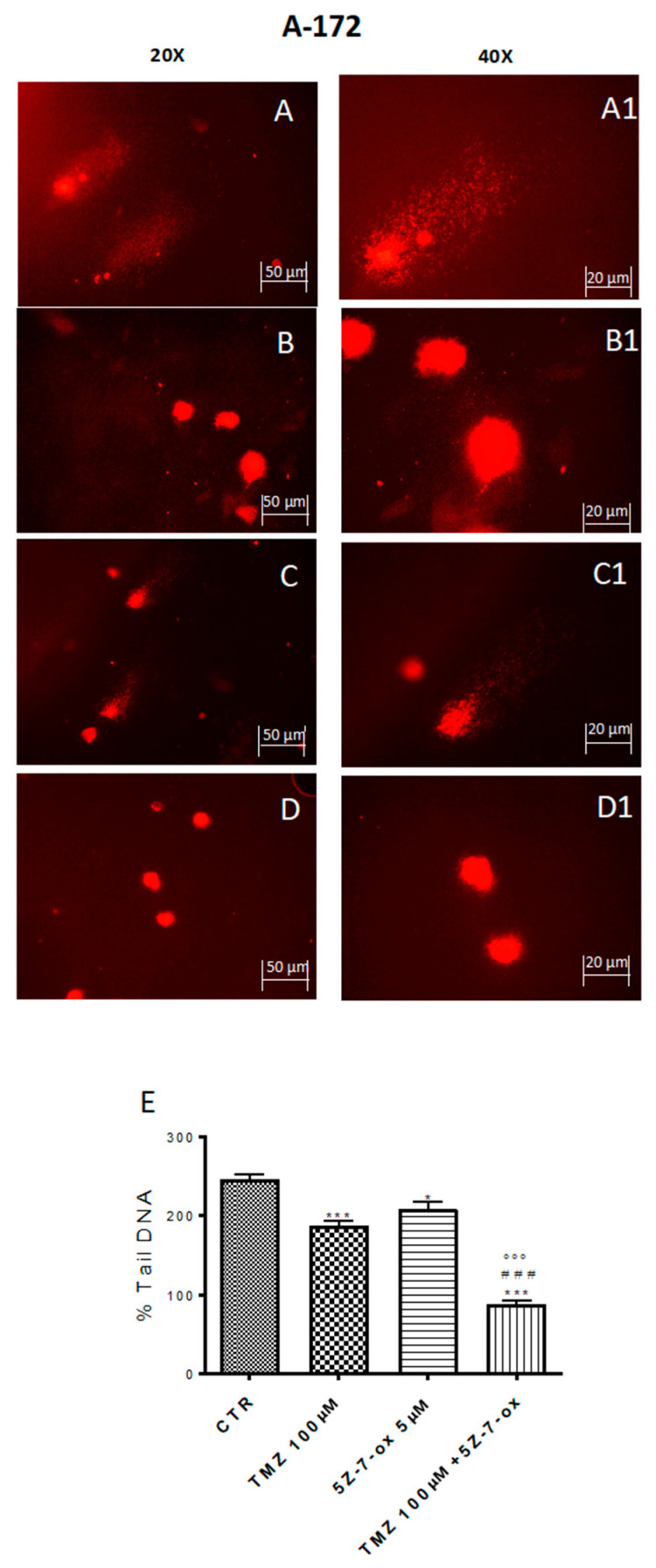
Effect of 5Z-7-ox and TMZ on DNA damage in A172 cell line. Alkaline comet assays (% tail DNA as comet pattern) were taken to quantify DNA damage. Panel (**A**) and densitometry in (**E**) shows the comet patterns obtained in glioblastoma control group, whereas panel (**B**,**C**) shows the comet pattern recorded from pre-incubation with TMZ and 5Z-7-ox indicating that pre-treatment was efficacious in reducing DNA damage, however panel (**D**) demonstrates how the association significantly reduced the % of DNA tail. (**A1**–**D1**) shows the high magnification at 40×. The graph (**E**) shows the densitometry analysis of comet patterns. Data are representative of at least three independent experiments. *** *p* < 0.001 vs. CTR, ### *p* < 0.001 vs. TMZ and °°° *p* < 0.001 vs. 5Z-7-ox.

**Figure 4:**
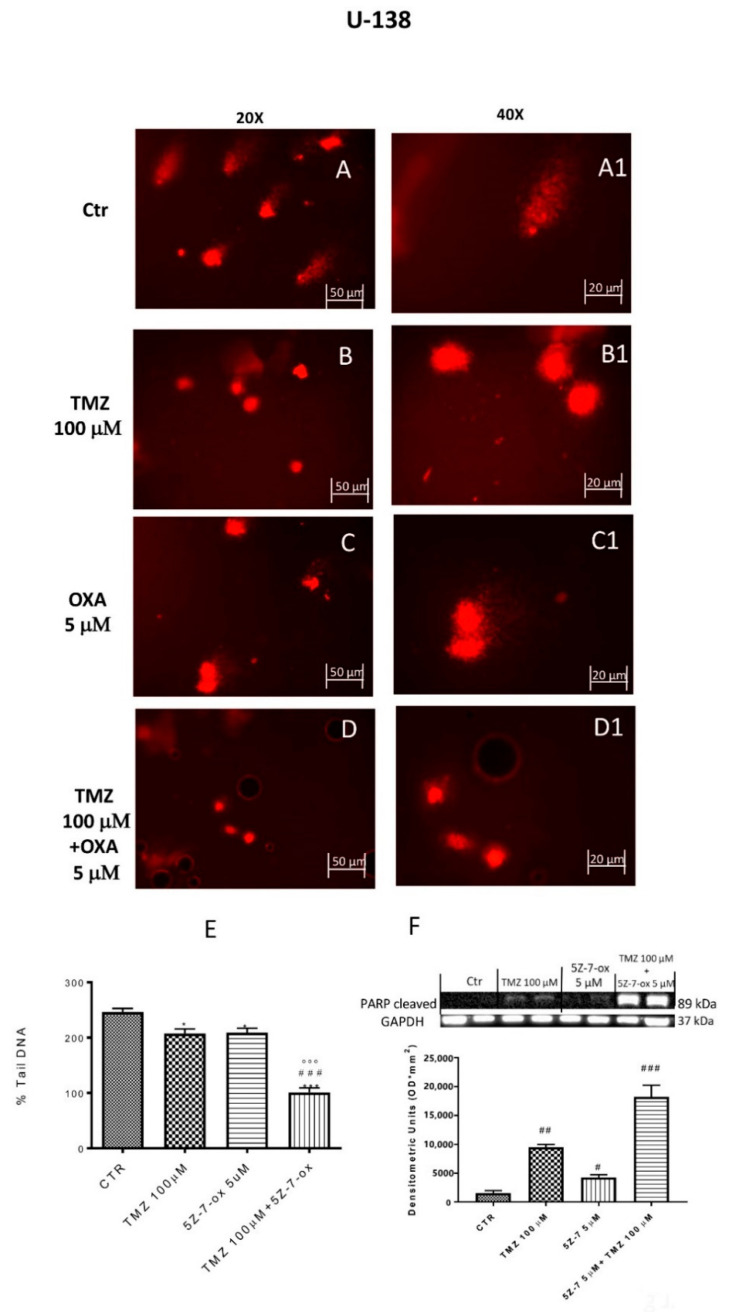
Effect of 5Z-7-ox and TMZ on DNA damage in U138 cell line. Alkaline comet assays (% tail DNA as comet pattern) were taken to quantify DNA damage. Panel (**A**) and densitometry in **F** shows the comet patterns obtained in glioblastoma control group, whereas panel (**B**,**C**) shows the comet pattern recorded from pre-incubation with TMZ and 5Z-7-ox showed that pre-treatment was efficacious in reducing DNA damage, however panel (**D**) demonstrates how the association significantly reduced the % of DNA tail. (**A1**–**D1**) shows the high magnification at 40×. The panel (**E**) shows the densitometry analysis of comet patterns. Data are representative of at least three independent experiments. * *p* < 0.05 vs. CTR, *** *p* < 0.001 vs. CTR, ### *p* < 0.001 vs. TMZ and °°° *p* < 0.001 vs. 5Z-7-ox. Panel **F** shows cleaved PARP-1 expression by Western blot analysis. A basal level of cleaved PARP in the CTR group cell lysates was observed, while the association between TMZ with 5Z-7-ox significantly up-regulated the expression of cleaved PARP-1 (**F**). Data are representative of at least three independent experiments. # *p* <0.05 vs. CTR, ## *p* <0.01 vs. CTR; ### *p* <0.001 vs. CTR. Original blots are shown in Figure S3.

DNA damage/repair system defects are the underlying molecular events driving the tumor initiation and progression [33]. The DNA repair capacity of TAK1 inhibitor, in association with TMZ, was evaluated by comet assay. Figure 3 and Figure 4 (A–D) show a spreading of DNA migration among control and treated cells including the measurement of the change in % tail DNA as a comet pattern. We observed a remarkable augmentation of DNA damage in the glioblastoma control cells (Figure 3 and Figure 4A, see % Tail DNA graph 3E). Moreover, by comet assay we assumed that TMZ was able to produce DNA damage in glioma cells (Figure 3 and Figure 4B, see % Tail DNA graph 3E). The comet patterns from the cells treated with 5Z-7-Ox 5 μM, associated with TMZ 100 μM (Figure 3 and Figure 4D, see % Tail DNA graph 3E), showed intact heads and a significant decrease in % of damaged DNA tail much more than components alone, representing an important protection from DNA loss (Figure 3 and Figure 4, panels B and C, see % Tail DNA graph 3E) (Figure 3E, F(3;4;7) = 103.9, *p* < 0.001 (Figure 4E, F(3;12;15) = 60.92, *p* < 0.001. Moreover, to confirm our observations, we evaluated—by Western blot analysis—the cleaved nuclear enzyme poly (ADP-ribose) polymerase (cPARP). The activation of PARP leads to extended chains of ADP-ribose to nuclear proteins leading to a substantial depletion of intracellular NAD and subsequently ATP, bringing cellular dysfunction until cell death [34]. Indeed, PARP-1 is proteolytically cleaved at the onset of apoptosis by caspases [35]. As expected, Western blot analysis showed an over expression of c PARP following 5Z-7-Ox 5 μM + TMZ 100 μM stimulation in U138 cell line (Figure 4F, F(3;4;7) = 95.67, *p* < 0.001, see densitometry analysis).

### 2.5. TAK1 Inhibited TMZ- Induced NF-κB Translocation 

**Figure 5:**
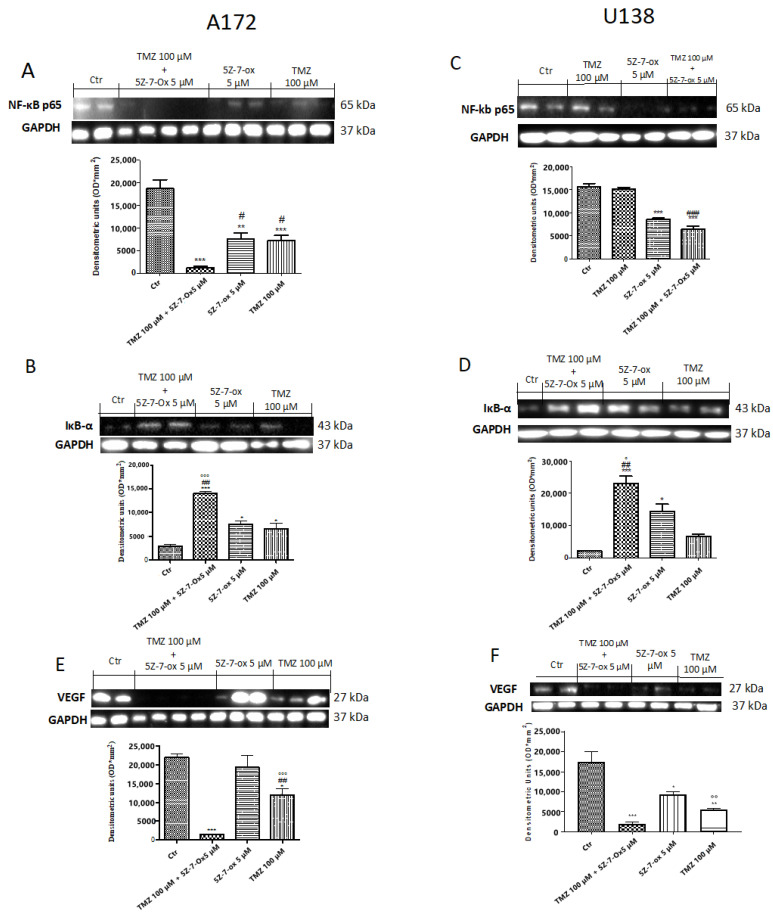
Effect of association (5Z-7-ox + TMZ) on Nf-kB pathway in glioblastoma cell lines. A low expression of IκBα was detected in the CTR group, whereas stimulation with 5Z-7-Ox 5 μM and TMZ 100 μM for 24 h partially increased IκBα levels (**B**) (A172) and (**D**) (U138). The association of TMZ with 5Z-7-ox considerably reduced IkBα degradation much more than single components ((**B**) (A172) and (**D**) (U138)). Moreover, NF-κB p65 expression was increased in the CTR group while the association significantly decreased its expression ((**A**) (A172) and (**C**) (U138)) compared to TMZ alone. Data are representative of at least three independent experiments. (**A**) *** *p* < 0.001 vs. CTR, ** *p* < 0.01 vs. CTR, # *p* < 0.05 vs. TMZ; (**B**) *** *p* < 0.001 vs. CTR, * *p* < 0.05 vs. CTR, ## *p* < 0.01 vs. TMZ and °°° *p* < 0.001 vs. 5Z-7-ox; (**C**) *** *p* < 0.001 vs. CTR and ### *p* < 0.001 vs. TMZ; (**D**) *** *p* < 0.001 vs. CTR, * *p* < 0.001 vs. CTR, ## *p* < 0.01 vs. TMZ and ° *p* < 0.05 vs. 5Z-7-ox. Western blot analysis showed a significant increase in VEGF expression in glioblastoma control cells ((**E**) (A172) and (**F**) (U138), see densitometry analysis, respectively), while the association between 5Z-7-Ox and TMZ significantly down-regulated its expression ((**E**) (A172) (**F**) (U138), see densitometry analysis, respectively) much more than single components ((**E**) (A172) (**F**) (U138), see densitometry analysis, respectively). Data are representative of at least three independent experiments. Data are representative of at least three independent experiments. (**E**) *** *p* < 0.001 vs. CTR, * *p* < 0.05 vs. CTR, ## *p* < 0.01 vs. TMZ, °°° *p* < 0.001 vs. 5Z-7-ox; (**F**) *** *p* < 0.001 vs. CTR, ** *p* < 0.01 vs. CTR, * *p* < 0.05 vs. CTR, °° *p* < 0.01 vs. 5Z-7-ox. Original blots are shown in Figures S4 and S5.

According to the identification that TAK1 is a required element for genotypic stress-induced NF-κB pathway activation [32], we hypothesized that the combinatory effect of 5Z-7-oxozeaenol with TMZ might be determined by inhibition of NF-κB activation. Then, to confirm this hypothesis, we studied whether TAK1 inhibition could reduce chemotherapy-induced NF-κB pathway activation in A172 and U138 cells. Basal level of IκBα was identified in the control group, while after 24 h, stimulation with 5Z-7-Ox (5 μM) and TMZ (100 μM) partially increased IκBα expression in both A172 and U138 cell lines (Figure 5B,F (3;8;11) = 37, *p* < 0.001; Figure 5D,F (3;7;11) = 26.27, 0.001; see densitometry analysis). The association of TMZ with TAK1 inhibitor significantly reduced IkBα degradation much more than single components (Figure 5B of A172 cell line, and Figure 5D of U138 cell line, see densitometric analysis). Furthermore, nuclear translocation of NF-κB p65 was higher in the control (CTR) group, while the association significantly decreased its expression (Figure 5A of A172 cell line, Figure 5F (3;8;11) = 38.76, *p* < 0.001; Figure 5C of U138 cell line, Figure 5F (3;8;11) = 65.52, *p* < 0.001, compared to the TMZ group.

### 2.6. TAK1 Inhibition Reduced Angiogenesis in Association with TMZ

To investigate whether the TAK inhibitor and TMZ association could stimulate normalization of the impaired neurovascular unit, we looked at vascular endothelial growth factor (VEGF) expression. Western blot analysis showed a significant increase in VEGF expression in glioblastoma control cells (Figure 5E (A172) and Figure 5F (U138), see densitometry analysis, respectively), while the association between 5Z-7-Ox and TMZ significantly down-regulated its expression (Figure 5E of A172 cell line, Figure 5F (3;9;12) = 32.31, *p* < 0.001; Figure 5F of U138 cell line, Figure 5F (3;4;7) = 91.39, *p* < 0.001; see densitometry analysis, respectively) much more than single components.

### 2.7. Role of 5Z-7-Ox on Tumor Growth in In Vivo Study

**Figure 6:**
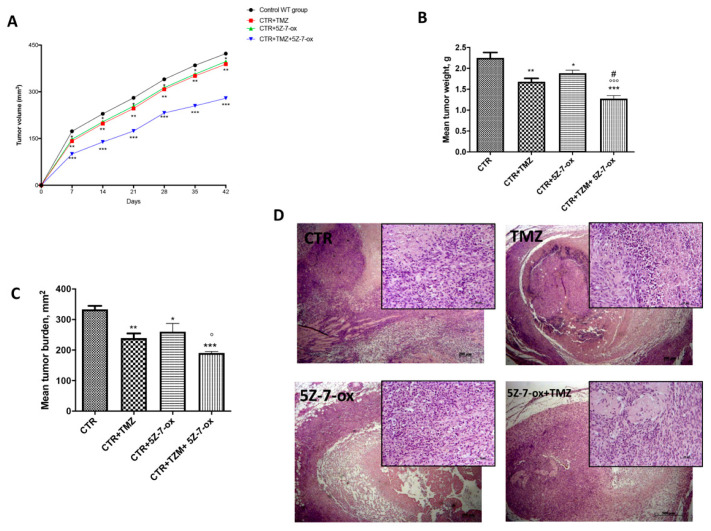
Effect of 5Z-7-Ox on tumor growth in the in vivo study. The panel (**A**) and (**B**) showed a significant reduction in tumor volume and weight, respectively. The panel (**C**) demonstrated that the association of 5Z-7-Ox and TMZ conferred a greater resistance to the tumor onset and expansion. As shown in panel (**D**), the association between TMZ and 5Z-7-Ox significantly reduced tumor sections as well as neutrophil infiltration, much more than single components. Data are representative of at least three independent experiments. (**A**) * *p* < 0.05 vs. CTR; ** *p* < 0.01 vs. CTR; *** *p* < 0.001 vs. CTR. (**B**) ** *p* < 0.01 vs. CTR; * *p* < 0.05 vs. CTR; *** *p* < 0.001 vs. CTR; °°° *p* < 0.001 vs. CTR+5Z-7-Ox; # *p* < 0.05 vs. CTR+TMZ (**C**) ** *p* < 0.01 vs. CTR; * *p* < 0.05 vs. CTR; *** *p* <0.001 vs. CTR; ° *p* < 0.05 vs. CTR+5Z-7-Ox.

**Figure 7:**
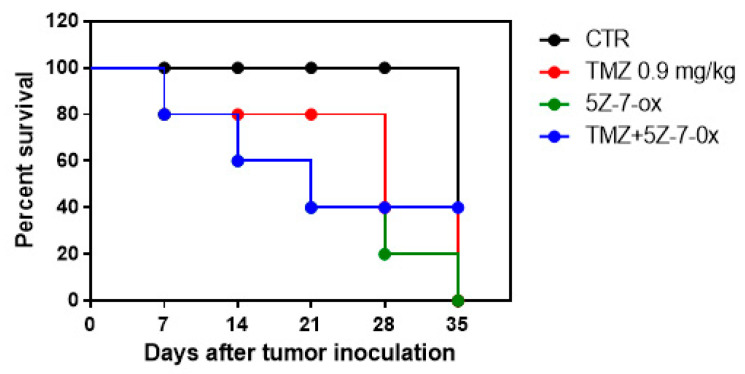
Survival curve in vivo. Kaplan–Meier survival analysis of C57BL/6 wild-type mice implanted with U87MG cells.

In this study, we observed that the association of 5Z-7-Ox with TMZ strongly inhibited tumor growth, as well as also significantly reducing tumor weight (Figure 6A,B, F(3;20;23) = 19.13, *p* < 0.001. An interesting result was seen in tumor burden (Figure 6C, F(3;20;23) = 12.52, *p* < 0.001, that was inhibited by 57%, suggesting that the association conferred a greater resistance to the tumor onset and progression. The histological analysis (Figure 6D) of the control group revealed a subcutaneous mass, composed of solid sheets and irregular round epithelioid cells ill-defined cell borders, as well as an increase in tumor necrosis and neutrophilic permeation; the association between TMZ and 5Z-7-Ox showed a significant reduction in tumor sections as well as neutrophil infiltration, much more than single components. The survival curves in vivo were calculated using the Kaplan–Meier method, as presented in Figure 7. No significance difference was seen between groups.

### 2.8. Effective Role of TAK1, MAPKs and NF- κB in Patients with GMB

**Figure 8:**
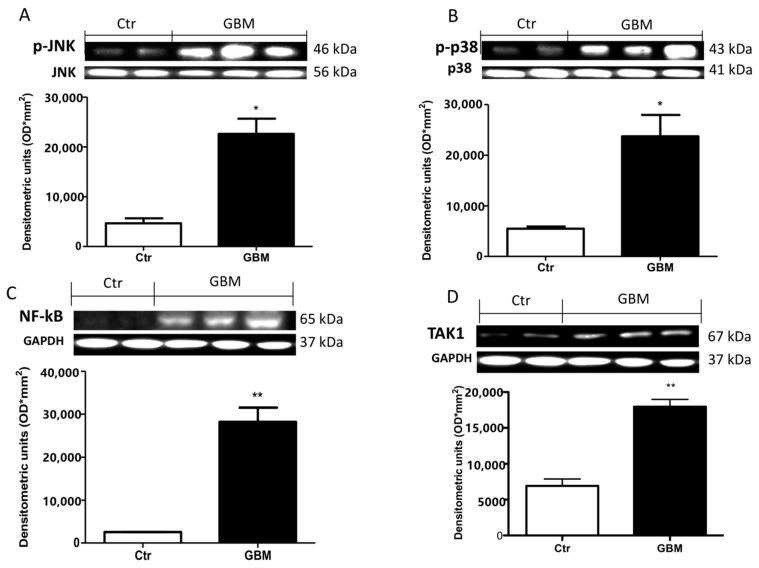
Effect of 5Z-7-ox and TMZ in mitogen-activated protein kinase (MAPK) kinase pathway and NfkB in patients. A substantial increase in p-JNK (**A**) and p-p38 (**B**) expression in glioblastoma patients compared to the control group was observed by Western blot analysis. Panel C shows an increase in NF-κB translocation in the GBM patients sample compared to patients in the control group (**C**). Panel (**D**) shows a notable up-regulation of TAk1 in GBM patients. Data are representative of at least three independent experiments. (**A**,**B**) from the same brain samples * *p* < 0.05 vs. CTR. (**C**,**D**) ** *p* < 0.01 vs. CTR. Original blots are shown in Figure S6.

To validate the efficacious use of the TAK1 inhibitor in patients affected by the recurrence of glioblastoma, we performed a Western blot analysis in glioblastoma TMZ treated tissue samples and compared this with brain tissue from healthy patients. Our results showed a significant up-regulation in phospho- c-Jun N-terminal kinase (p-JNK) (Figure 8A, see densitometry analysis) and p-p38 (Figure 8B, see densitometry analysis) in glioblastoma TMZ-treated patients compared to the control group (Figure 8A,B, respectively, see densitometry analyses), denoting the continuous involvement of MAPKs in tumorigenesis following chemotherapy. Moreover, an important increase in NF-κB translocation was visible in GMB TMZ-treated patients compared to patients control group (Figure 8C, see densitometry analysis). Our hypothesis was strongly validated by an evident up-regulation of TAK-1 in GMB patients (Figure 8D, see densitometry analysis).

## 3. Discussion

GBM remains the primary malignancy of the CNS. Despite relevant progress in the research of novel therapies, the prognosis is still tragic; therefore, the development of compounds to collectively target different pathways involved in the progression of gliomagenesis is needed. Understanding the molecular mechanisms that mediate the chemo-resistance of neoplastic cells in GBM is critically essential for curing patients affected by GBM. Since it is well established that TAK1 plays a critical role in chemotherapy-induced NF-κB activation [32], we hypothesized that the association between an inhibitor of TAK1 and TMZ in GMB could potentiate the efficacy of these conventional chemotherapeutic agents, even leading to reduce the inflammatory pathways.

GBM is one of the most highly angiogenic solid tumors [36]. GBM alters healthy tissue vasculature, remodeling vessel network, increasing diameter and thickening membranes. This “singular” cancer vasculature is supposed to enhance tumor hypoxia and slow the distribution of chemotherapies [37,38]. These mechanisms lead to the activation of different factors such as the regulator of angiogenesis VEGF [39,40], considered an attractive target to exploit in cancer therapeutics [41]. Our data evidenced the upregulation of VEGF in glioblastoma cell lines. Chemotherapy treatment with TMZ reduced its expression. However, the association with a TAK1 inhibitor significantly decreased this factor associated with tumor vascularization. Additionally, vascular defective permeability, an altered apoptotic program is recurrent in GBM cells, which evade death through the alterations of the p53 pathway and the inhibition of pro-apoptotic Bcl-2 members. Several growth factor pathways involved in the regulation of apoptosis, can represent possible ways in which GBM cells could be targets for new therapies [42]. Our results showed an important alteration of apoptotic proteins that were increased by chemotherapy treatment. However, the association of TMZ with 5Z-7-oxozeaenol significantly increased pro apoptotic protein levels, much more than TMZ alone and identifying these cells still inclined towards therapy-induced apoptosis.

Regarding the tumors, the DNA damage/repair system possess two distinguished aspects: first, it defends the integrity of genetic material of healthy cells; second, it contributes to the resistance of tumor-driving cells to therapies [33]. At the beginning of gliomagenesis, the DNA damage response (DDR) mechanism is activated by oncogene replication and/or oxidative stress [43], working as a protective machinery; however, during their growing, cancer cells can accumulate and tolerate genome damage because of DDR anomalies. PARP1 is a sensor of single-strand breaks (SSBs) involved in several systems of DNA repair [44]. The overexpression of PARP1 has been found in different cancers including GBM; indeed, it has been demonstrated that PARP inhibitors, alone or in combination with chemotherapeutic agents, induced promising effects against tumors harboring DNA repair defects [45].

First and foremost, our results confirmed relevant DNA damage and PARP up-regulation in glioblastoma cell lines. However, the combination between TMZ and the TAK1 inhibitor significantly reduced DNA tail fragments and the expression of PARP1, highlighting this association as a respectable DNA repair modulator.

NF-κB is a transcription factor regulating a large number of activities related to cellular functions, including proliferation differentiation and cell survival. Different studies defined the aberrant activation of NF-κB as a frequent event in cancer progression, including GMB. [46]. In addition, NF-κB has been linked with TMZ resistance primarily due to the anti-apoptotic activity of NF-κB [47]. Thanks to the association with the TAK 1 inhibitor, 5Z-7-oxeazeanol, TMZ could markedly reduce the activation of the NF-κB pathway. Moreover, the U87-xenograft model showed that 5Z-7-ox associated with TMZ produced a moderate tumor volume inhibition, as well as a remarkable reduction in tumor growth.

Present-day management for GBM implies the combination of chemotherapy and radiation therapy (RT); this combination increases the overall survival of GBM patients; however, TMZ and RT combination is effective for a limited time and GBM patients maintain a poor overall survival rate [48]. Here, it is proven that the combination of pathway inhibitors and TMZ or RT, might be a successful strategy for the treatment of GBM.

## 4. Materials and Methods

### 4.1. In Vitro Studies

#### 4.1.1. Cell Lines

The human GBM cell lines U138MG (U-138 MG ATCC^®^ HTB-16™ Homo sapiens brain glioblastoma IV grade), A172 (A-172 (A172)\ATCC^®^ CRL-1620™ Homo sapiens brain glioblastoma), U87 MG (ATCC^®^ HTB-14™ Homo sapiens brain, likely glioblastoma IV grade) and L229 (LN-229 ATCC^®^ CRL-2611™ Homo sapiens brain/right frontal parieto-occipital cortex glioblastoma IV grade) were purchased from American Type Culture Collection (ATCC) (Manassas, VA, USA). The GMB human cell lines U138MG, A-172, U87 and L229 were seeded in 75 cm^2^ flask, respectively: U138 with ATCC-formulated Eagle’s Minimum Essential Medium (Catalog No. 30-2003) supplemented with fetal bovine serum to a final concentration of 10% and A172, U87 and L229 with ATCC-formulated Dulbecco’s Modified Eagle’s Medium (Catalog No. 30-2002) supplemented with fetal bovine serum (concentration of 10%) at 37 °C in 5% CO2.

#### 4.1.2. Cell Viability Assay (Preliminary Study).

The cellular viability of GBM cell lines was assessed using a mitochondria-dependent dye for living cells (tetrazolium dye; MTT) to formazan, as previously described [49]. Cultures were pre-treated with increasing concentrations of 5Z-7-ox (0,1–5 μM) and TMZ (1–100 μM) and were then incubated at 37 °C with MTT (0.2 mg/mL) for 1 h, to determine high concentrations with high toxicity on cell viability. The extent of reduction of MTT to formazan was measured by optical density (550 nm) with a microplate rider.

Once chosen the effective concentration of TMZ and 5Z-7-ox, cell cultures were divided into 4 groups (9 well/group):

1. CTR: U138, A172, U87 and L229 cultured with normal culture medium.
2. CTR + TMZ 100 μM: cultures were treated for 24 h with TMZ at a concentration of 100 μM
3. CTR + 5Z-7-oxozeaenol (5Z-7-ox): 5 μM: cultures were treated for 24h with 5Z-7 ox at a concentration of 5 μM
4. CTR + association TMZ 100 μM and 5Z-7ox 5 μM: cultures were treated for 24 h with 5Z-7 ox and TMZ at a concentration of 5 μM and 100 μM, respectively.

#### 4.1.3. Cell Viability Assay to Test the Efficacy of Association

The cellular viability of A-172, U-138, U87 and L229 cells was evaluated using a mitochondria-dependent dye for living cells (tetrazolium dye; MTT) to formazan, as previously described [37]. Cultures were treated with 5Z-7-oxozeaenol (5 μM), temozolomide (100 μM) and the association between them (TMZ 100 μM and 5Z-7ox 5 μM).

#### 4.1.4. Western Blot Analysis

Western blot analysis in U138MG and A-172 cell lines was conducted according to [49]. The expression of IKBα, nuclear factor kappa-light-chain-enhancer of activated B cells (NFκB), Bax, poli ADP-ribose polymerase (PARP1), caspase 3, and vascular endothelial growth factor (VEGF) was quantified in total cells proteins. The filters were probed with specific Abs: anti- IKBα (1:500, Santa Cruz Biotechnology, sc 1643, Paso Robles, CA, USA), anti- NF-κB (1:1000, BD Transduction Laboratories, Lexington, KY, USA), anti-Caspase 3 (1:500; Santa Cruz Biotechnology, sc 7272), anti-Bax (1:500; Santa Cruz Biotechnology, sc 7480), anti-cleaved PARP (cPARP) (1:500; Santa Cruz Biotechnology, sc-56196) and anti-VEGF (1:500; Santa Cruz Biotechnology, sc 7269). Glyceraldehyde-3-Phosphate Dehydrogenase (GAPDH) (1:1000, Santa Cruz Biotechnology, sc-47724), c-Jun N-terminal kinases (JNK) (1:500, Santa Cruz Biotechnology, sc 6254) and mitogen-activated protein kinases (p38) (1:1000, Santa Cruz Biotechnology), were used to confirm equal protein loading and blotting.

#### 4.1.5. Alkaline (pH > 13) Comet Assay

The alkaline microgel electrophoresis or “comet assay” determined the extent of cellular DNA damage. It was performed in A172 and U138 cells, as previously described [50]

The percentage of DNA in the comet tail (%TDNA) was used as a DNA damage parameter. Results were stated as tail length, which is an indication of the presence of DNA damage, expressed as mean of the 50 cells scored.

#### 4.1.6. Statistical Evaluation for In Vitro Study

All values, in the figures, were evaluated as mean ± S.E.M. A total of 9 wells/group were used and the experiments were conducted in triplicate. The results were examined by one-way analysis of variance followed by a Bonferroni post hoc test for multiple comparisons. A *p*-value of <0.05 was considered significant.

### 4.2. In Vivo Studies

#### 4.2.1. Cell Line 

Human glioblastoma cell line U-87 MG (U-87 MG ATCC^®^ HTB-14™ Homo sapiens brain, likely glioblastoma) was obtained from ATCC (American Type Culture Collection, Rockville, MD, USA). U-87 MG cells were cultured in 75 cm^2^ flasks with ATCC-complete culture medium available as ATCC^®^ Catalog No. 4-X.

#### 4.2.2. Animals

Wild-type mice—4–16-week-old male C57BL/6J—were purchased from Jackson Laboratory (Bar Harbor, Hancock, ME, USA) and housed in microisolator cages under pathogen-free conditions on a 12 h light/12 h dark schedule for a week. Food and water were available ad libitum. This study was approved by the University of Messina Review Board for the care of animals, in compliance with Italian regulations on protection of animals (n° 368/2019-PR released on 14 May 2019). Animal care was in accordance with Italian regulations on the use of animals for the experiment (D.M.116192) as well as with the Council Regulation regulations (EEC) (O.J. of E.C. L 358/1 12/18/1986). 

#### 4.2.3. Experimental Design

Tumor induction was performed, as previously described [51]. After being housed for a week, the mice were inoculated subcutaneously with 3 × 10^6^ glioblastoma U-87 MG cells in 0.2 mL PBS and 0.1 mL Matrigel (BD Bioscience, Bedford, MA). After injection, the mice were monitored for forty-two days, then the animals were sacrificed and their tumors were excised and processed for histological analysis. Dimensions (length and width) of tumors were measured using a digital caliper, and the tumor burden was calculated using the following formula: 0.5 × length × width. The mean weight of the mice at the initiation of the study and termination of the study did not differ significantly between the groups. The tumor size was measured every four days for 28 days. The tumor volume was calculated using an empirical formula, V = 1/2 × ((the shortest diameter)2 × (the longest diameter)).

Experimental groups:

1. Group 1 (vehicle): weekly intravenous (IV) administration of physiologic solution (7, 14, 21, 28, 35 days.)
2. Group 2: daily oral (OS) administration of TMZ 0.9 mg/kg, from day 7 to day 35.
3. Group 3: daily intraperitoneal (ip) administration of 5Z-7-ox 15mg/kg, from day 7 to day 35.
4. Group 4: TMZ-5Z-7-ox combination from day 7 to day 35.

The experimental design, cell line chosen and substance doses were based on previous studies [32,52,53].

#### 4.2.4. Histology

Mice were sacrificed at 42 days after U87 injection and tumors were processed for histology via staining with hematoxylin and eosin. Tumor sample sections of 7 μm thickness were processed and evaluated by a qualified histopathologist. All sections were studied using an Axiovision Zeiss microscope (Milan, Italy).

#### 4.2.5. In Vivo Survival Curve 

Survival curves were determined by the Kaplan–Meier method. Mice were considered expired when the tumor volume reached 0.0025 L post-treatment in accordance with the Institutional Animal Care and Use Committee regulations [54]. At the end of the experiment (day 35), the survival of the control group and experimental groups were all sacrificed.

#### 4.2.6. Statistical Analysis

All values, in the figures, were evaluated as mean ± SEM. The results were analyzed by ANOVA one-way analysis of variance, followed by a Bonferroni post-hoc test for multiple comparisons; *p* values <0.05 were considered statistically significant.

### 4.3. Clinical Studies

#### 4.3.1. Patients and Samples

This study involved 30 cases of patients affected by recurrence of glioblastoma, including 21 men and 9 women aged between 40 and 65 years. The patients underwent a second surgery between January and July 2017. All patients were operated on at the Unit of Neurosurgery at the University of Messina (ME, Sicily, Italy). Recurrence of brain tumors and tissues removed during surgery in patients with severe head injury operated on for large brain contusions were collected during craniotomies. The tissues were immediately stored at −80 °C before the experimental study. Histopathologic evaluation of brain tumor tissues was performed by expert neuropathologists based on the World Health Organization Grade 4 classification of gliomas. Neuropathological and biomolecular analysis revealed that all operated tumors were recurrence GBMs from patients treated with Stupp protocol and adjuvant chemotherapy with temozolomide. All patients provided informed consent for both tumor and healthy brain collection, that was approved by the Regional Ethical Board at the University of Messina. By reviewing patient records and contacting patients, families and referring physicians, all the necessary information for the study was gathered (age, sex, clinical presentation and surgical data, number of cycles of temozolomide, neuroradiological data).

Tissue samples were divided into 2 groups:

1. Control group (CTR): healthy brain tissues used as negative control removed during surgery in patients with severe head injury operated on for large brain contusions (*n* = 30)
2. GBM samples: recurrence of glioblastoma tissues from patients treated with TMZ used as positive control (*n* = 30)

#### 4.3.2. Western Blot Analysis

Protein extraction and Western blot analyses in brain tissue were performed as previously described [55,56]. The expression of NF-κB, p-JNK and p-p38 was quantified in total tissue proteins. The filters were probed with specific Abs: anti- NF-κB (1:500; Santa Cruz Biotechnology), anti-p-JNK (1:500; Santa Cruz Biotechnology), p-p38 (1:500; Abcam, Cambridge, MA, USA) or and anti- TAK1 (1:1000; Cell Signaling, Danvers, MA, USA). P38 (1:500, Santa Cruz Biotechnology), JNK (1:500, Santa Cruz Biotechnology) and GAPDH (1:2000, Santa Cruz Biotechnology) were used to confirm equal protein loading and blotting.

### 4.4. Materials

Unless otherwise stated, all compounds were obtained from Sigma-Aldrich. All other chemicals were of the highest commercial grade available. 5Z-7-oxozeaenol was acquired from Tocris Bioscience. All stock solutions were produced in non-pyrogenic saline (0.9% NaCl, Baxter, Milan, Italy). 

### 4.5. Statistical Analysis

All values, in the figures, were evaluated as mean ± SEM. The results were analyzed by ANOVA one-way analysis of variance followed by a Bonferroni post-hoc test for multiple comparisons and Student’s *t*-test to compare two groups (unpaired test); *p-*values < 0.05 were considered statistically significant.

## 5. Conclusions

The poor prognosis and the high recurrence of GBM represents an urgent clinical issue and reinforces the necessity to explore and to develop novel therapeutic approaches that are associated with the clinical guidelines of TMZ or RT. Therefore, specific cancer molecular targets are expected to have a beneficial effect to increase the efficacy of common therapies [57]. Our results are compatible with the evidence that the combination of TAK inhibitors and TMZ might be an effective strategy for the treatment of GBM, showing capacity to sensitize GMB tumor cells to chemotherapeutic agents. Moreover, our data could demonstrate the tangible involvement of TAK1 and MAPKs in GMB patients undergoing chemotherapy, supporting our hypothesis. In this era of targeted therapy, our findings provide a novel therapeutic approach indicating TAK1 inhibition as a promising target into the cultivated fields of cancer research.

## Data Availability

The data presented in this study are available on request from the corresponding author.

## Supplementary Materials

The following are available online at https://www.mdpi.com/2072-6694/13/1/41/s1, Figure S1: Original blots A172 of Figure 2, Figure S2: Original blots U138 of Figure 2, Figure S3: Original blots of Figure 4, Figure S4: Original blots A172 of Figure 5, Figure S5: Original blots U138 of Figure 5; Figure S6, Original blots of Figure 8.
