# Supplementary material for: TAK1 Inhibitor Enhances the Therapeutic Treatment for Glioblastoma
Source: Cancers (Basel). 2020 Dec 25;13(1):41. doi: 10.3390/cancers13010041 (PMC7794959; doi:10.3390/cancers13010041)
Supplement: Supplementary file 1 [file cancers-13-00041-s001.pdf]

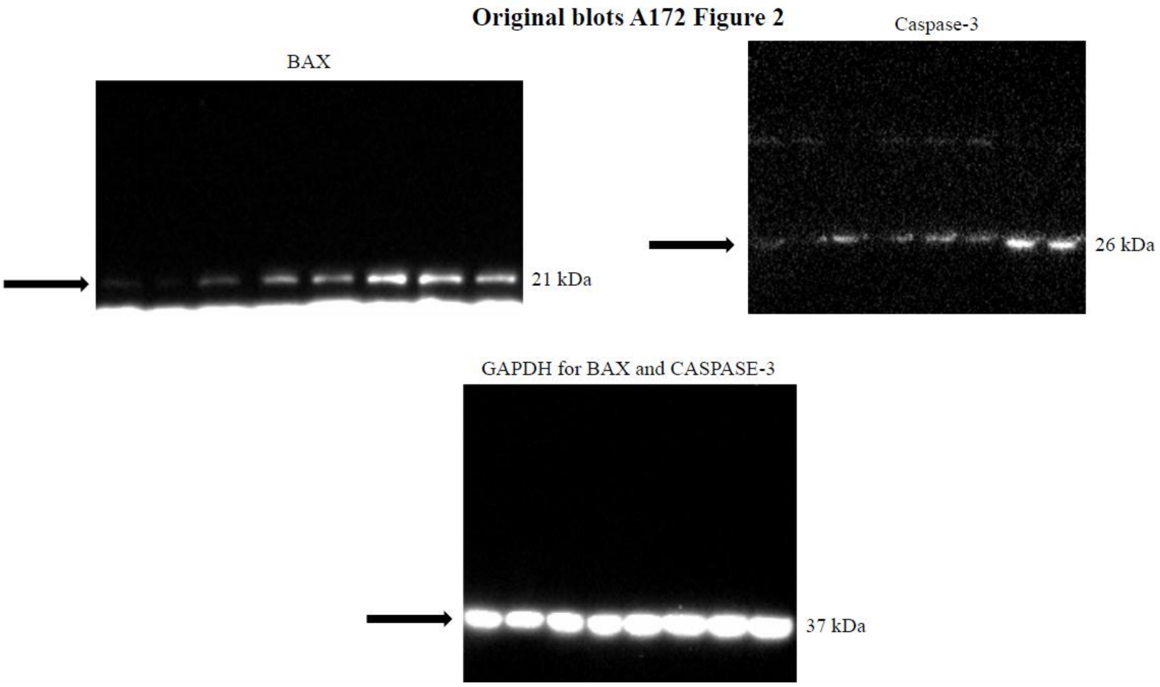

Figure S1. Original blots A172 of Figure 2.

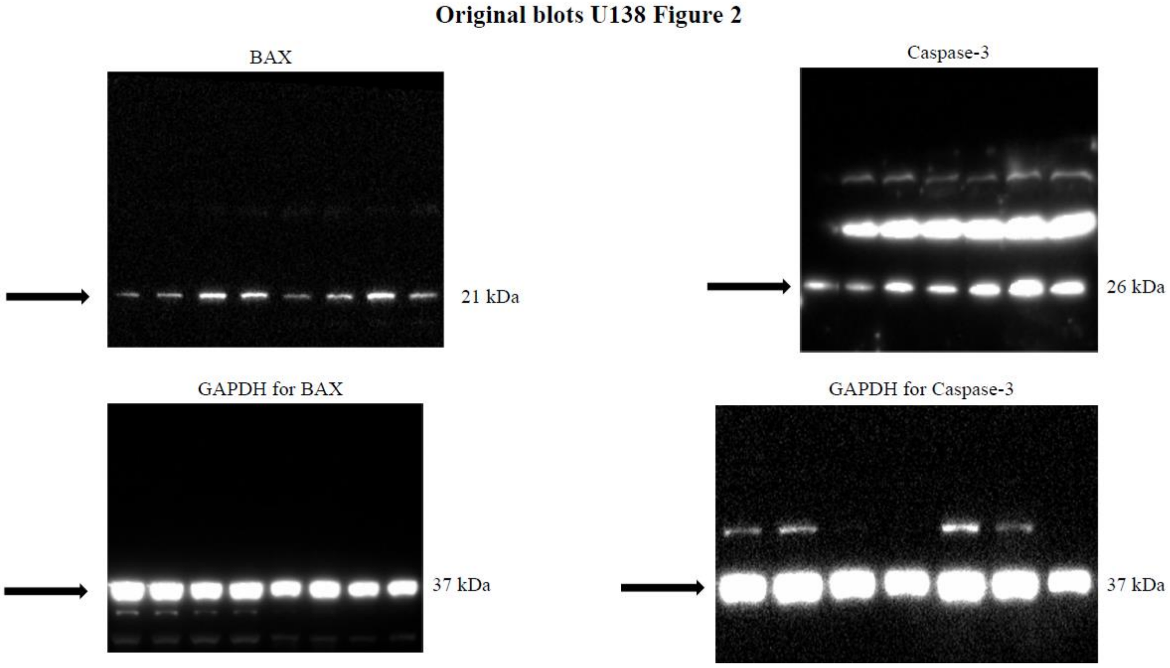

Figure S2. Original blots U138 of Figure 2.

Original blots Figure 4

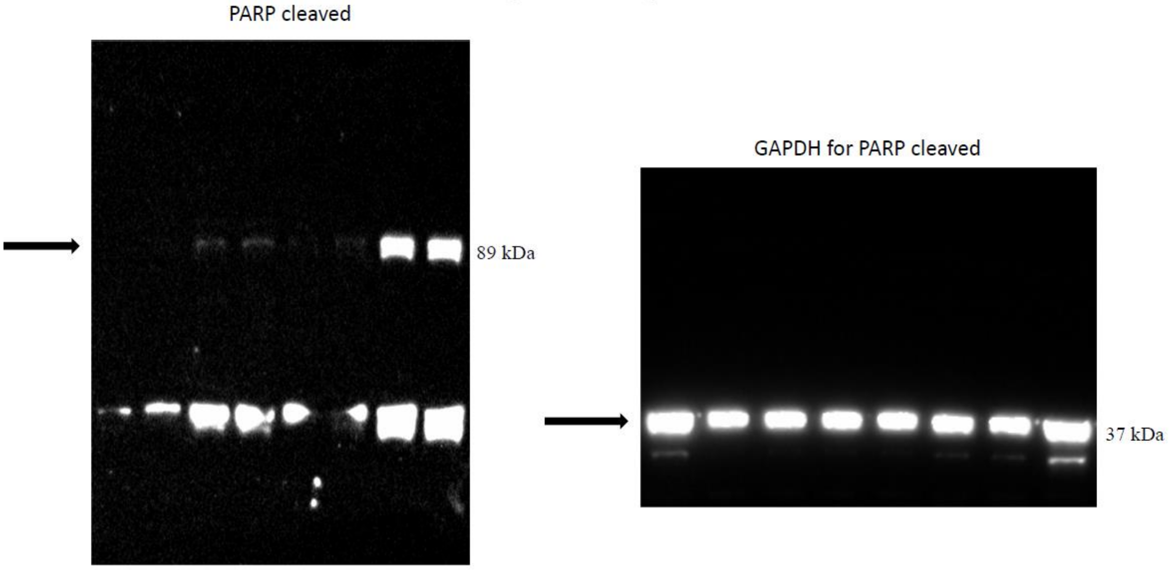

Figure S3. Original blots of Figure 4.

Original blots A172 Figure 5

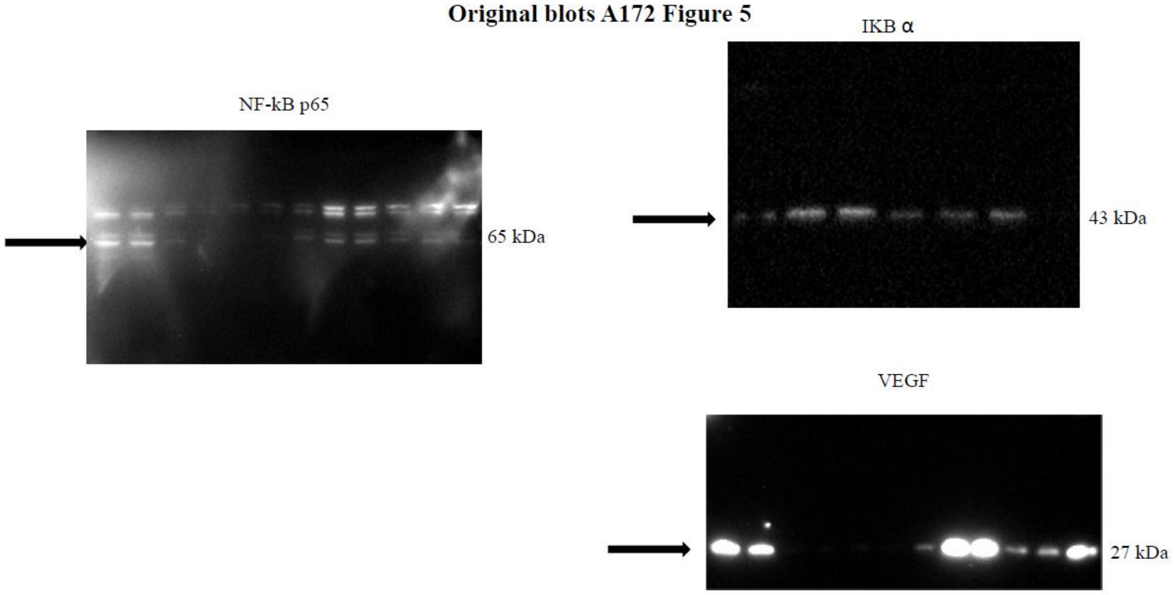

Original blots A172 Figure 5

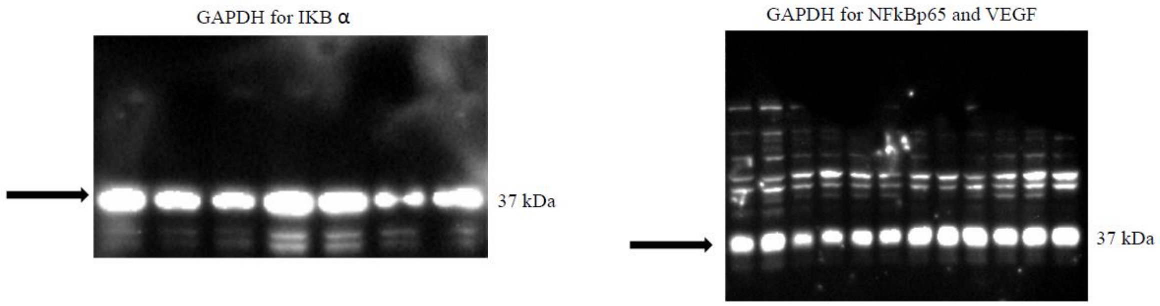

Figure S4. Original blots A172 of Figure 5.

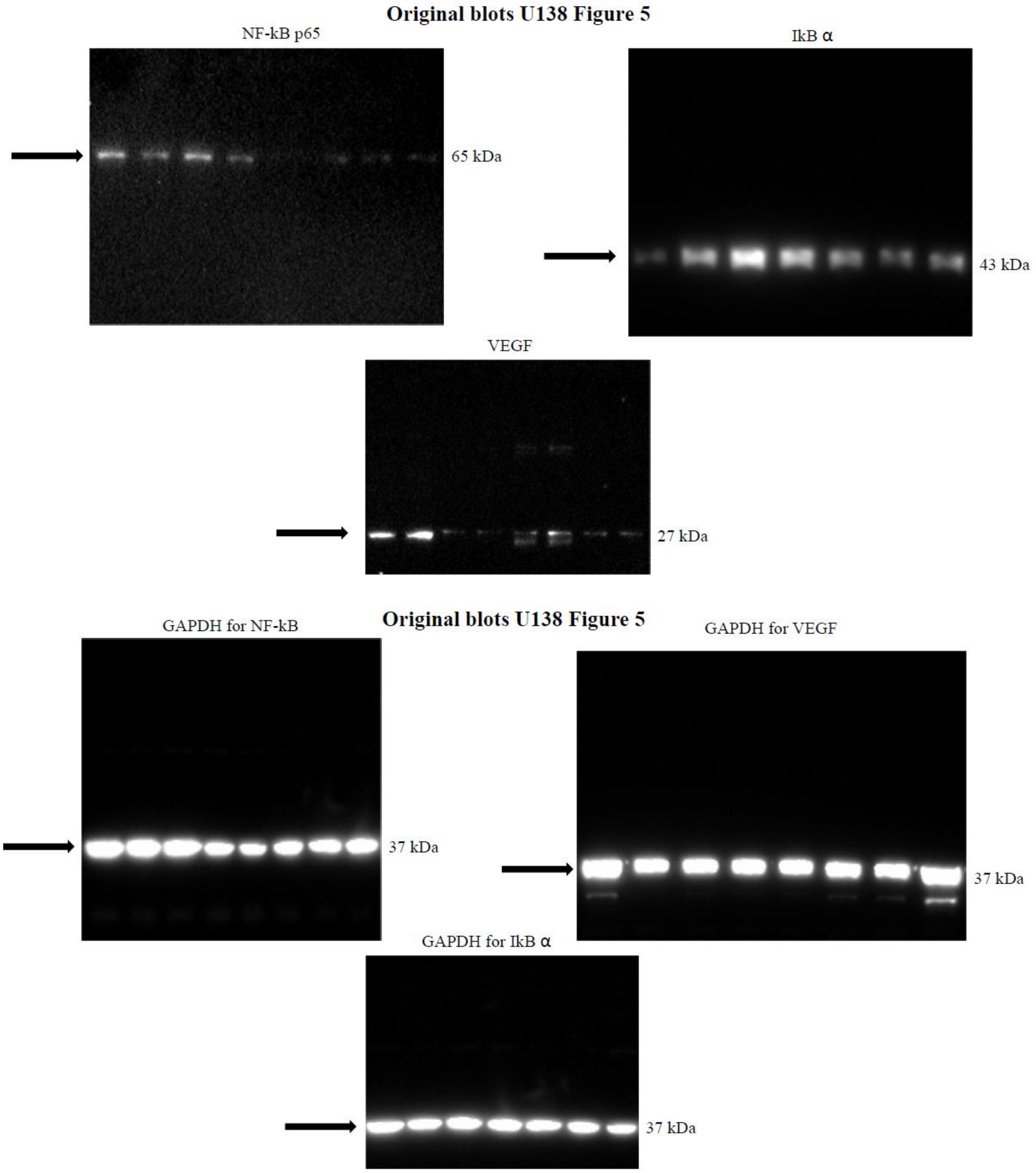

Figure S5. Original blots U138 of Figure 5.

Original blots Patients Figure 8

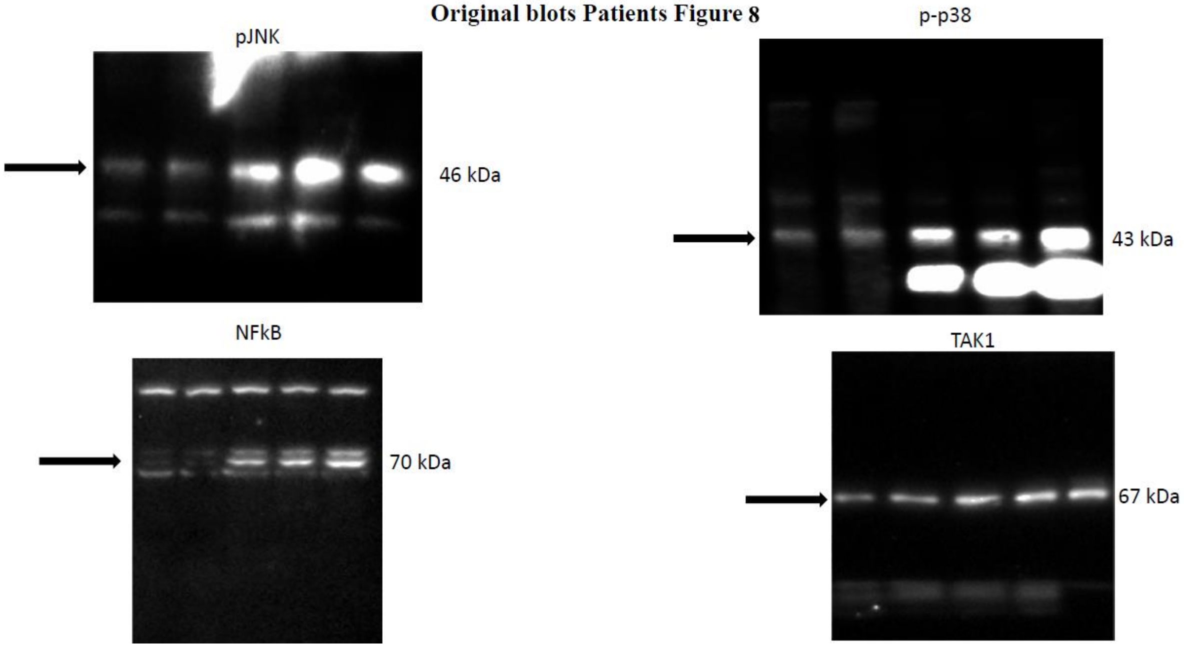

Original blots Patients Figure 8

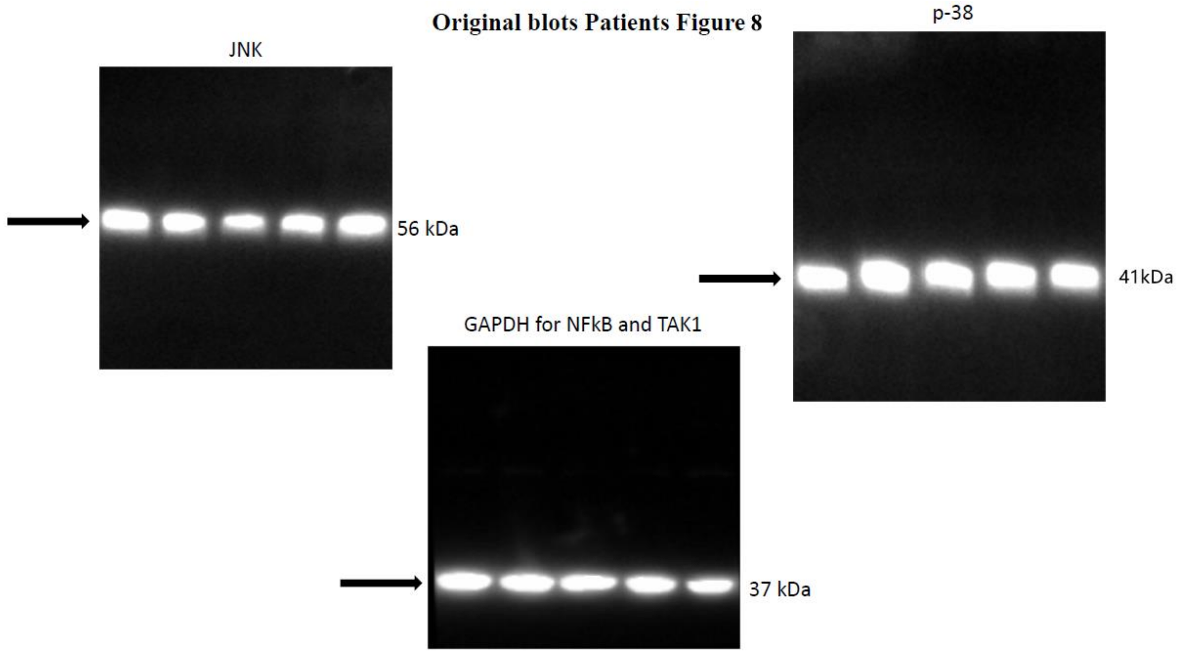

Figure S6. Original blots of Figure 8.
